# Around the Hospitals

**Published:** 1903-04-18

**Authors:** 


### AROUND THE HOSPITALS.

### CITY OF LONDON LYING-IN HOSPITAL.

The medical report gives the usual particulars of the cases admitted; there were 524 patients, of whom three died; 505 infants were born alive, 27 were still-born. The figures are below those of the preceding six years; this is believed to be due to a rumour that the hospital was closed or about to be closed. Nineteen infants died, the majority from prematurity. There was one case of cleft spine. There were 2,242 cases in the out-patient department, this being the largest number in any one year since the establishment of this branch in 1872. Eight hundred and eight women were attended in their own homes, by the midwives and nurse-pupils, under the supervision of the outdoor midwife, and in all cases of doubt and difficulty with the further assistance of the district medical officers. There were no deaths from puerperal causes.

### CHARING CROSS HOSPITAL: THE YEAR'S WORK.

The hospital was open throughout 1902, and in spite of the difficulty of carrying on the work during alterations, the resident and nursing staff performed their duties in a quiet and uncomplaining manner, not infrequently under conditions of great discomfort. There were 20,059 new patients, viz., 1,986 in-patients and 18,073 out-patients, including 8,929 cases of accident and emergency. The total attendances were 44,772. The ordinary income, including subscriptions and donations (£2,319 and £1,802), boxes, Hospital Sunday Fund (£1,045) and Hospital Saturday Fund (£267), Hughes estate, dividends, miscellaneous and rents, was £8,695 13s. 10d. Extraordinary receipts were £17,884 16s. 8d. The total ordinary expenditure, including £659 19s. 6d. for the medical school, was £15,306 17s. 7d. The sum of £32,320 was expended on the new buildings; £105 on heating apparatus, smoke consumer, etc.; and £319 on the medical school. These are under the head of extraordinary expenditure. The receipts for the general fund, including legacies, were £16,412; for the special appeal, £2,645; from the festival dinner, £5,022; for the convalescent home, £1,197. A grant of £2,500 for the improvement fund, and £100 for the convalescent home, was made by King Edward's Fund. The cost per bed is estimated at £85 11s., and of each in-patient £6 9s. Seats to view the Coronation Procession realised only £106. The number of patients admitted to the convalescent home was 538. The Lord Mayor made a grant of £50 from the Transvaal War Fund for the maintenance of 70 soldiers. Admission to the home, which is at Walton-on-Thames, is not restricted to patients from the hospital.

